# Triple-Negative Metaplastic Breast Carcinoma With Squamous and Glandular Differentiation Treated With Neoadjuvant Chemoimmunotherapy: A Case Report

**DOI:** 10.7759/cureus.111739

**Published:** 2026-06-29

**Authors:** Autumn Forde, Alexius Ramcharan

**Affiliations:** 1 Surgery, Harlem Hospital Center, New York, USA

**Keywords:** grandular differentiation, immunotherapy, invasive breast carcinoma, keynote-522, metaplastic breast carcinoma, neoadjuvant chemoimmunotherapy, pembrolizumab, rare breast tumor, squamous differentiation, triple negative metaplastic breast carcinoma

## Abstract

Metaplastic breast carcinoma with squamous and glandular differentiation is a rare and aggressive subtype of breast cancer that frequently exhibits a triple-negative phenotype and lacks standardized treatment guidelines. We report the case of a 69-year-old female who presented with a progressively enlarging left breast mass and a palpable axillary lymph node. Imaging demonstrated a suspicious 4 cm breast mass and an enlarged axillary lymph node with cortical thickening. Core needle biopsy revealed a high-grade invasive carcinoma with squamous and focal glandular differentiation, consistent with metaplastic breast carcinoma. Immunohistochemistry demonstrated a triple-negative phenotype with expression of CK5/6 and p63, supporting squamous differentiation and the diagnosis of metaplastic breast carcinoma. Axillary lymph node biopsy was negative for metastatic disease. The patient was initiated on neoadjuvant chemoimmunotherapy according to the KEYNOTE-522 regimen, with plans for breast-conserving surgery. This case highlights the diagnostic challenges and evolving treatment strategies for this rare breast cancer subtype.

## Introduction

Metaplastic breast carcinoma (MpBC) is a rare and heterogeneous subtype of invasive breast cancer, accounting for less than 1% of all breast malignancies. According to the World Health Organization classification, MpBC encompasses a diverse group of tumors characterized by differentiation of the neoplastic epithelium into squamous and/or mesenchymal elements. Tumors with squamous differentiation are particularly uncommon and may occur as pure squamous carcinomas or in combination with glandular components. Histologically, squamous differentiation is characterized by keratinization, intercellular bridges, and polygonal epithelial cells, whereas glandular differentiation retains features of conventional adenocarcinoma [1,2].

The diagnosis of MpBC requires careful histopathologic evaluation because its appearance may overlap with that of other entities, including high-grade invasive ductal carcinoma, primary cutaneous squamous cell carcinoma involving the breast, and metastatic squamous cell carcinoma from an extramammary site. Immunohistochemistry is often helpful in establishing the diagnosis. These tumors frequently express basal cytokeratins such as CK5/6 and p63 and may demonstrate positivity for breast lineage markers, including GATA3. Most cases exhibit a triple-negative immunophenotype characterized by absent estrogen receptor (ER), progesterone receptor (PR), and HER2 expression. This receptor profile limits the use of targeted therapies and is associated with aggressive clinical behavior and poorer outcomes compared with conventional invasive ductal carcinoma [2-4]. Beyond establishing the diagnosis, histopathologic and immunohistochemical findings may provide important prognostic information. Metaplastic carcinomas frequently exhibit high-grade morphology, elevated proliferative activity, and a triple-negative phenotype, all of which have been associated with increased recurrence risk and poorer survival compared with conventional invasive ductal carcinoma. Recognition of these features is therefore important for both diagnosis and treatment planning.

Patients with metaplastic carcinoma typically present with rapidly enlarging breast masses and may exhibit overlying skin changes or palpable axillary lymphadenopathy. Radiologic findings are often nonspecific and can resemble those of high-grade triple-negative invasive ductal carcinoma, making tissue diagnosis essential. Multiple retrospective studies have demonstrated lower pathologic complete response rates to conventional neoadjuvant chemotherapy, increased recurrence rates, and reduced overall survival compared with other triple-negative breast cancers [2,3,5]. More recently, immune checkpoint inhibitors have emerged as a promising treatment strategy in high-risk triple-negative breast cancer. Although data specific to MpBC remain limited, the frequent triple-negative phenotype and immunogenic characteristics of these tumors provide a rationale for incorporating chemoimmunotherapy into treatment approaches [6,7]. We present a case of triple-negative MpBC with squamous and glandular differentiation treated with neoadjuvant chemoimmunotherapy according to the KEYNOTE-522 regimen.

## Case presentation

A 69-year-old woman presented to the breast clinic with a progressively enlarging left breast mass that had been present for approximately one year. She reported mild tenderness and localized erythema over the affected area but denied nipple discharge, fever, unintentional weight loss, or other constitutional symptoms. Her medical history was otherwise unremarkable. She denied tobacco use and had no known family history of breast or ovarian cancer. Physical examination revealed a firm, irregular 4 × 4 cm mass located at the 11 o'clock position of the left breast, approximately 7 cm from the nipple. A palpable left axillary lymph node measuring approximately 1.5 cm was also identified.

**Figure 1:**
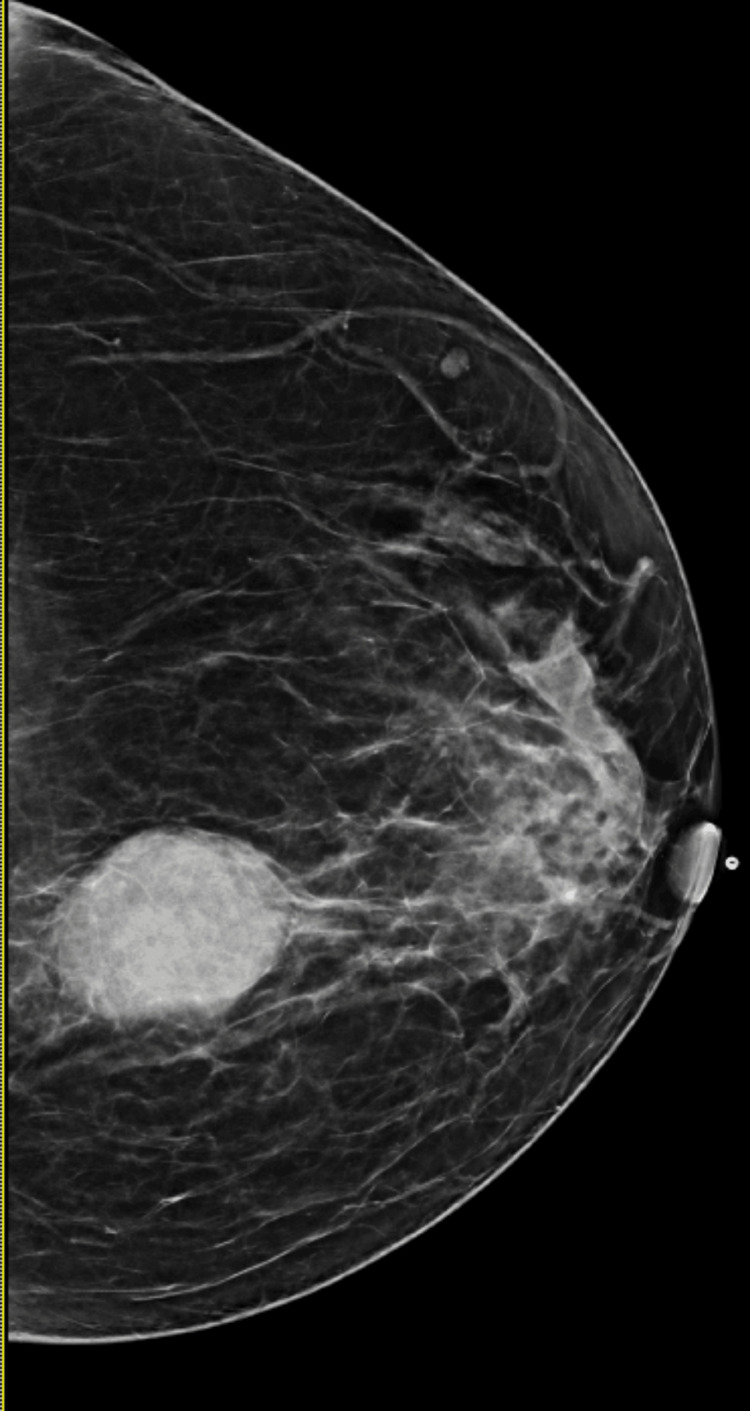
Diagnostic mammography demonstrating a 4 cm irregular high-density mass with indistinct margins in the upper inner quadrant of the left breast.

**Figure 2:**
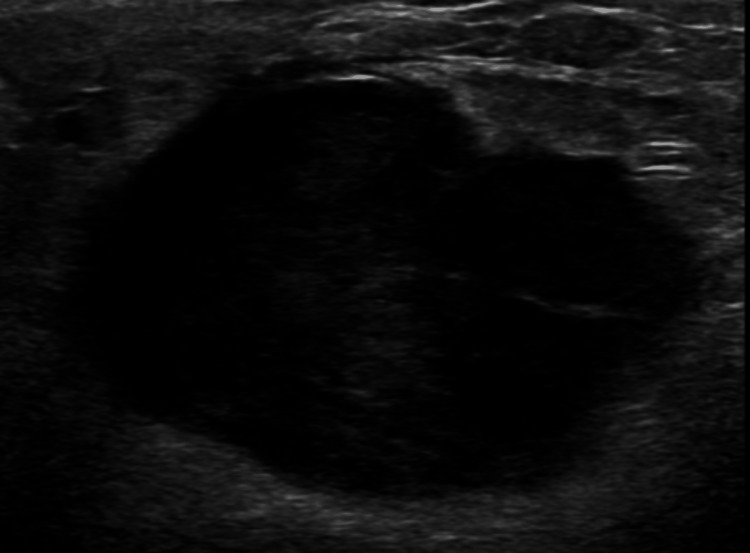
Targeted ultrasound showing a 4 cm irregular, mixed echogenic mass located 7 cm from the nipple corresponding to the palpable mass.

Diagnostic mammography demonstrated a 4 cm irregular, high-density mass with indistinct margins in the upper inner quadrant of the left breast (Figure 1). Targeted ultrasound confirmed a corresponding irregular mixed-echogenicity lesion (Figure 2). Ultrasound evaluation of the left axilla demonstrated a 1.4 cm lymph node with cortical thickening, raising concern for nodal involvement.

**Figure 3:**
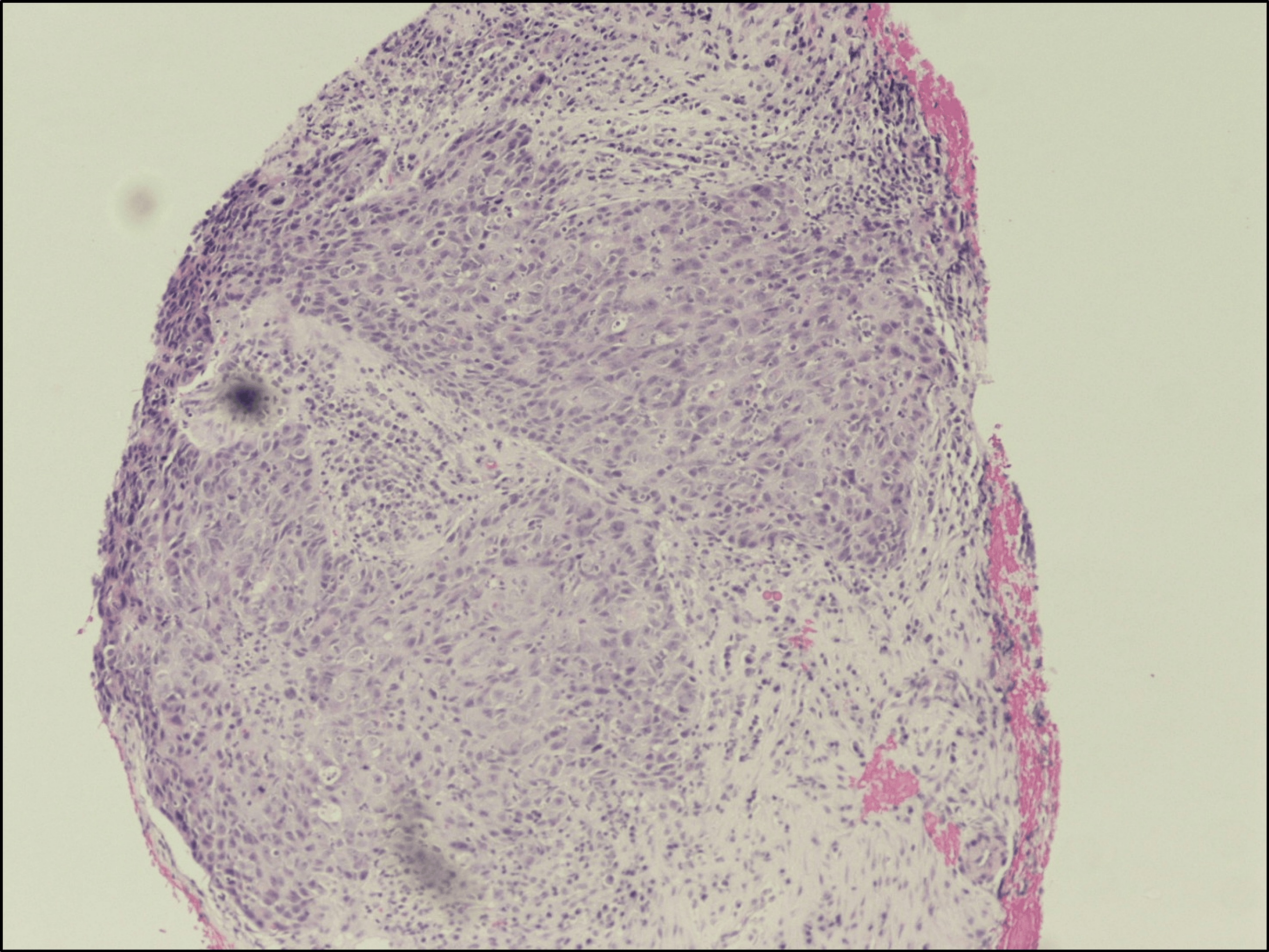
Hematoxylin and eosin (H&E) stain (10×) demonstrating high-grade invasive carcinoma with squamous and focal glandular differentiation, consistent with metaplastic breast carcinoma.

**Figure 4:**
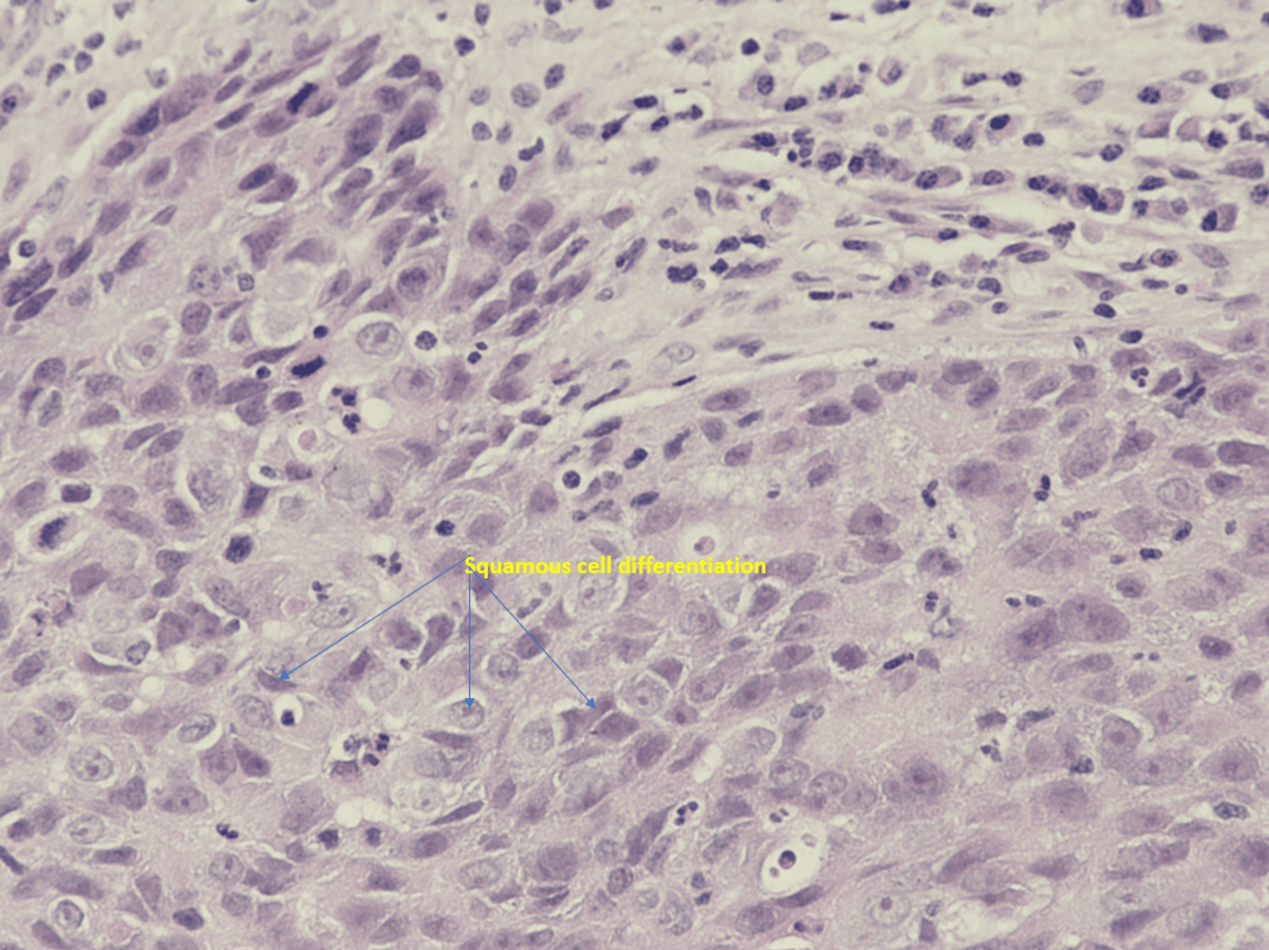
H&E stain (40×) demonstrating squamous differentiation characterized by keratinization and intercellular bridges (arrows).

**Figure 5:**
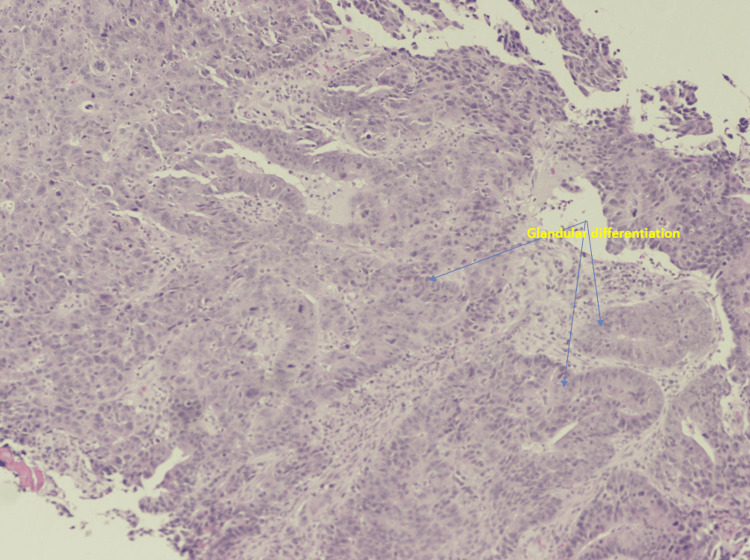
H&E stain (10×) demonstrating focal glandular differentiation within the metaplastic carcinoma (arrows).

**Figure 6:**
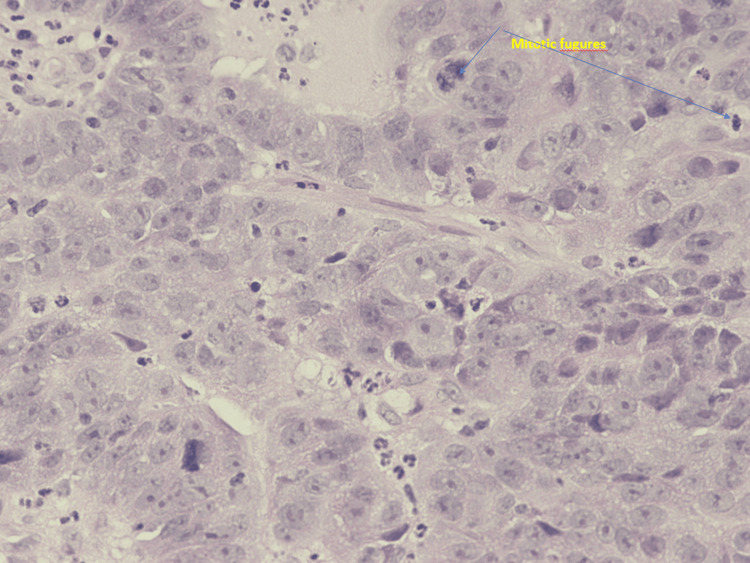
H&E stain (40×) demonstrating frequent mitotic figures (arrows), supporting the high-grade morphology of the tumor.

**Figure 7:**
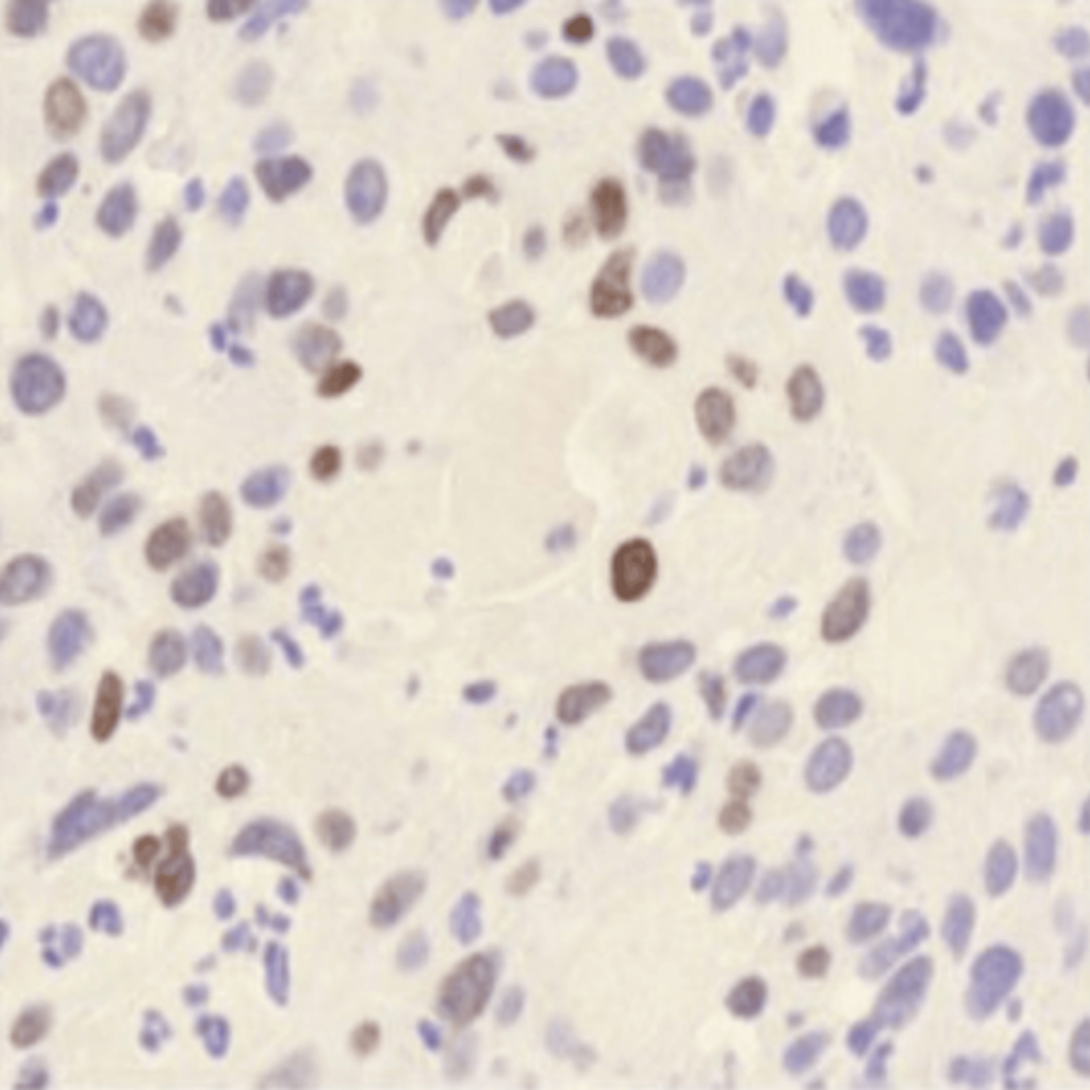
GATA3 immunohistochemical stain demonstrating weak and focal expression, supporting mammary origin in triple-negative metaplastic breast carcinoma with squamous and focal glandular differentiation.

Ultrasound-guided core needle biopsy of the breast mass demonstrated a high-grade invasive carcinoma with squamous and focal glandular differentiation, consistent with MpBC (Figures 3-7). Histologic examination demonstrated characteristic squamous differentiation with keratinization and intercellular bridges, focal glandular differentiation, and frequent mitotic figures, consistent with a high-grade neoplasm. Immunohistochemical analysis demonstrated negative ER and PR expression and HER2 negativity according to current CAP/ASCO criteria. Additional immunohistochemical studies demonstrated expression of AE1/AE3, CK7, CK5/6, and p63, with focal expression of CK14, S100, and weak focal GATA3 expression (Figure 7). Ki-67 immunostaining demonstrated high proliferative activity. The morphologic and immunophenotypic findings supported the diagnosis of triple-negative MpBC with squamous and focal glandular differentiation. 

Staging evaluation revealed no evidence of distant metastatic disease. The patient was initiated on neoadjuvant chemoimmunotherapy according to the KEYNOTE-522 regimen, consisting of pembrolizumab in combination with carboplatin and paclitaxel, followed by anthracycline-based chemotherapy. Plans were made for subsequent breast-conserving surgery with sentinel lymph node biopsy following completion of systemic therapy.

## Discussion

MpBC with squamous and glandular differentiation is a biologically distinct and aggressive subtype of breast cancer associated with poorer outcomes compared with conventional invasive ductal carcinoma and other triple-negative breast cancers of similar stage [2-5]. The rarity of the disease has limited the development of evidence-based treatment guidelines, and management is frequently extrapolated from studies involving conventional triple-negative breast cancer.

The pathologic diagnosis of metaplastic breast carcinoma with squamous and glandular differentiation can be challenging because it overlaps with several entities, including high-grade invasive ductal carcinoma with squamoid features, primary cutaneous squamous cell carcinoma involving the breast, and metastatic squamous cell carcinoma from an extramammary site. Histologic features such as keratinization and intercellular bridges favor true squamous differentiation, while the presence of glandular elements supports a diagnosis of metaplastic carcinoma rather than pure squamous cell carcinoma. In the present case, histologic examination demonstrated characteristic squamous differentiation with keratinization and intercellular bridges, focal glandular differentiation, and frequent mitotic figures (Figures 3-6), supporting the diagnosis of a high-grade metaplastic carcinoma. Immunohistochemistry further supported the diagnosis, as metaplastic carcinomas frequently express basal cytokeratins such as CK5/6 and squamous markers, including p63, while maintaining a predominantly triple-negative phenotype. Consistent with these findings, the tumor demonstrated expression of CK5/6 and p63, together with focal CK14 and weak focal GATA3 expression (Figure 7). These findings supported a mammary epithelial origin and helped distinguish this tumor from other carcinomas with squamoid features and metastatic squamous malignancies.

Several retrospective studies have reported lower response rates to conventional neoadjuvant chemotherapy in metaplastic breast carcinoma compared with conventional triple-negative breast cancer. Pathologic complete response rates as low as 10%-20% have been described, suggesting relative chemoresistance in this histologic subtype [3,5]. Consequently, the identification of novel treatment approaches remains an area of active investigation.

The KEYNOTE-522 trial demonstrated that the addition of pembrolizumab to neoadjuvant chemotherapy significantly improved pathologic complete response rates and event-free survival in patients with high-risk early-stage triple-negative breast cancer [6,7]. Although metaplastic breast carcinoma was not specifically evaluated as a separate subgroup, the frequent triple-negative phenotype and immunogenic characteristics of these tumors provide a biologic rationale for incorporating immune checkpoint inhibition into treatment strategies. The present case illustrates the application of neoadjuvant chemoimmunotherapy in a patient with triple-negative metaplastic breast carcinoma with the goal of maximizing tumor regression and facilitating breast-conserving surgery.

## Conclusions

MpBC with squamous and glandular differentiation is a rare, high-grade malignancy with aggressive clinical behavior and limited data to guide treatment. Early histopathologic diagnosis is critical because these tumors may mimic conventional triple-negative breast cancers in both imaging and clinical presentation. Given the historically poor response to standard chemotherapy and the frequent triple-negative immunophenotype, neoadjuvant chemoimmunotherapy represents a rational strategy to promote tumor regression and facilitate breast-conserving surgery, although further studies are needed to determine its impact on outcomes. Prognosis remains uncertain, underscoring the importance of careful follow-up and continued reporting of similar cases. This case highlights the need to recognize uncommon histologic subtypes of breast cancer, apply emerging therapies thoughtfully, and expand the clinical evidence base to better inform management and optimize outcomes for patients with MpBC.
